# Home-made enzymatic premix and Illumina sequencing allow for one-step Gibson assembly and verification of virus infectious clones

**DOI:** 10.1186/s42483-020-00077-4

**Published:** 2020-12-03

**Authors:** Mingmin Zhao, Beatriz García, Araiz Gallo, Ioannis E. Tzanetakis, Carmen Simón-Mateo, Juan Antonio García, Fabio Pasin

**Affiliations:** 1 Centro Nacional de Biotecnología (CNB-CSIC), 28049 Madrid, Spain; 2 College of Horticulture and Plant Protection, Inner Mongolia Agricultural University, Hohhot 010018, China; 3 Department of Entomology and Plant Pathology, Division of Agriculture, University of Arkansas System, 72701 Fayetteville, USA; 4 University of Padova, 35122 Padova, Italy

**Keywords:** Virus infectious clone, Agro-infection, Gibson assembly, Illumina sequencing, Home-made cloning reagents, *Potyvirus*, Tobacco vein mottling virus

## Abstract

An unprecedented number of viruses have been discovered by leveraging advances in high-throughput sequencing. Infectious clone technology is a universal approach that facilitates the study of biology and role in disease of viruses. In recent years homology-based cloning methods such as Gibson assembly have been used to generate virus infectious clones. We detail herein the preparation of home-made cloning materials for Gibson assembly. The home-made materials were used in one-step generation of the infectious cDNA clone of a plant RNA virus into a T-DNA binary vector. The clone was verified by a single Illumina reaction and a de novo read assembly approach that required no primer walking, custom primers or reference sequences. Clone infectivity was finally confirmed by *Agrobacterium*-mediated delivery to host plants. We anticipate that the convenient home-made materials, one-step cloning and Illumina verification strategies described herein will accelerate characterization of viruses and their role in disease development.

## Background

Viruses have major economic and societal impacts. As the main causes of emerging infectious diseases, viruses are major threats to human health and global food security (Anderson et al. 2004; Jones and Naidu 2019; Petersen et al. 2020). High-throughput sequencing is increasingly adopted to study disease etiology and has greatly improved our ability to detect and discover viruses, including in plants (Madroñero et al. 2019; Massart et al. 2019; Villamor et al. 2019; Ibaba and Gubba 2020). Yet, disease causality is difficult to confirm in the presence of mixed virus infections.

Infectious clone technology is a universal approach applied in disease causality studies and reverse genetics of viruses (Perez 2017; Pasin et al. 2019; Zang et al. 2020). Infectious clones have been proven powerful tools in the validation of metagenomics and high-throughput sequencing findings, extending our knowledge of virus genetics, establishing systems for biological characterization of viruses, and having myriad biotechnological applications (Grigoras et al. 2009; Ng et al. 2014; Jaschke et al. 2019; Pasin et al. 2019; Sicard et al. 2019; Abrahamian et al. 2020; Dinesh-Kumar and Voytas 2020; Xie et al. 2020).

First generation of infectious clones were generated using restriction enzymes for cloning copies of viral genomic sequences into plasmid vectors; lack of suitable restriction sites could, however, restrain cloning of large genomes. Homology-based cloning methods have been recently developed and used to generate infectious clones of plant viruses (Pasin et al. 2019), and viral agents of human and veterinary diseases (Siridechadilok et al. 2013; Almazán et al. 2014; Suhardiman et al. 2015; Teeravechyan et al. 2016; Ma et al. 2020). In vivo and in vitro homology-based methods were comprehensively reviewed elsewhere (Chao et al. 2015). They are efficient and flexible as they only require limited information of the virus sequence (e.g. a ~ 30-nt sequence of genomic termini). In vivo homology-based assembly is extremely efficient in yeast and has been used to construct > 100-kb infectious clones (Shang et al. 2017), and to engineer reverse genetic systems of plant viruses (Liang et al. 2004; Youssef et al. 2011; Desbiez et al. 2012; Sun et al. 2017). The low plasmid yields and growth rates of yeast are major limitations for routine cloning. In vitro homology-based methods compatible with routine cloning in *Escherichia coli* include In-Fusion (Takara Bio), GeneArt seamless cloning (Thermo Fisher Scientific), Gibson assembly (hereafter GA) and its derivative NEBuilder HiFi DNA assembly (New England Biolabs). These technologies have been successfully used to construct infectious clones of plant viruses (Blawid and Nagata 2015; Wieczorek et al. 2015; Laufer et al. 2018; Pasin et al. 2018; Feng et al. 2020), and have recently been reviewed (Pasin et al. 2019). Most commercial mixes for in vitro homology-based cloning are proprietary, undisclosed formulations that hamper scalability or adoption by scientists with limited resources.

In this study we report a convenient streamlined approach for one-step assembly and verification of infectious clones suitable for *Agrobacterium*-mediated delivery (agro-infection) to plants. The home-made materials and strategies described are compatible with routine cloning in *E. coli*. They rely on the efficiency and flexibility of the one-step isothermal DNA assembly method described by Gibson et al. (2009), a set of mini T-DNA binary vectors recently described (Pasin et al. 2017), and an automated clone verification approach using Illumina sequencing (Pasin et al. 2018). Finally, we applied the reagents and strategies presented to generate and verify a T-DNA binary vector including a full-length, intron-free cDNA clone of tobacco vein mottling virus (TVMV; genus *Potyvirus*) which was successfully delivered to plants by agro-infection.

## Results

### Preparation of the home-made enzymatic premix and *E. coli* competent cells for one-step Gibson assembly (GA)

Before commercial availability of mixes for the DNA assembly method described by Gibson et al. (2009), home-made GA reagents were used for infectious clone engineering and reverse genetic studies of plant viruses. Bordat et al. (2015) used home-made material to modify lettuce mosaic virus (LMV; genus *Potyvirus*) for the expression of a fluorescent protein gene. Home-made reagents were used by us to engineer a set of mutant and chimeric constructs of plum pox virus (PPV; genus *Potyvirus*) and investigate the P1-protease role in potyviral infections (Pasin et al. 2014, 2020; Shan et al. 2015). Based on protocols used for potyviral reverse genetics, reagents for GA were obtained from commercial providers or prepared in-house (Table 1). Reagent stock solutions were autoclaved or passed through a 0.22-μm filter as indicated (Tables 1 and 2). GA cofactors, enhancers and substrates (i.e. MgCl_2_, DTT, dNTP mix, NAD^+^, and PEG-8000) were combined in an aqueous solution buffered by Tris-HCl (pH 7.5) to a 3.33× reaction buffer (Table 2). Enzymes were then aliquoted to the reaction buffer for a 2× enzymatic premix with the DNA exonuclease, polymerase and ligase required for the one-step assembly and repair of the linear DNA molecules with overlapping termini (Table 3). The premix was aliquoted and stored at − 20 °C until needed.

Chemical transformation of bacterial cells does not require specialized equipment. Chemically-competent *E. coli* DH10B cells were prepared in-house according to the Hanahan’s procedure for high-efficiency transformation (Green and Sambrook 2018); transformation efficiencies was assessed, and lots giving ≥10^8^ transformants/μg of plasmid DNA were used in downstream experiments.

### One-step Gibson assembly of a potyvirid infectious clone using home-made cloning materials

We used tobacco vein mottling virus (TVMV), a member of the family *Potyviridae,* in the proof-of-concept experiments*. Potyviridae* is the largest family of plant RNA viruses, and members have large, single-stranded RNA genomes with an average size of ~ 9.7 kb (Wylie et al. 2017). Construction of potyvirid infectious clones is often technically challenging. It is a laborious process which may include multiple cloning steps and hosts (e.g. *E. coli* and yeast), whereas the clones may lack desirable features such as agro-infection capacity (Pasin et al. 2019). In addition, introns and cryptic promoter mutagenesis are often used to avoid toxicity and allow propagation of the infectious clones in bacteria (Johansen and Lund 2008; Tran et al. 2019; Klenov and Hudak 2021).

**Fig. 1:**
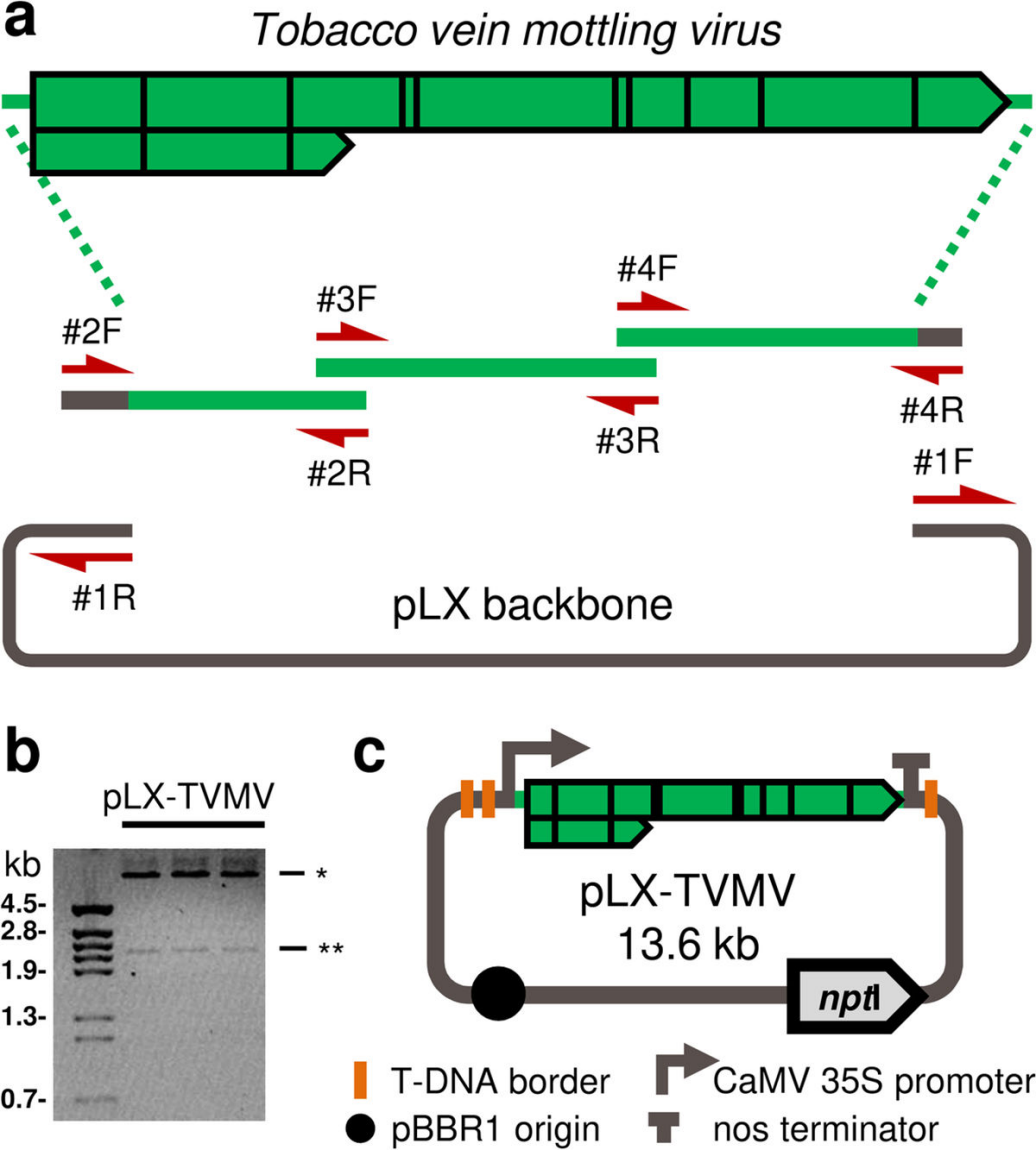
One-step assembly of a *Potyvirus* infectious clone by a home-made Gibson assembly enzymatic premix. **a** Genomic organization of tobacco vein mottling virus (TVMV) and cloning strategy. Three cDNA fragments spanning the TVMV genome were assembled into a linearized T-DNA binary vector (pLX backbone); the PCR primers used are indicated (red arrows). **b** Digestion profiles are shown of purified plasmids from selected *Escherichia coli* colonies. Left, DNA size marker; right, the *Xba*I-digestion pattern computed for pLX-TVMV is shown (**, a TVMV-specific fragment). **c** Diagram of pLX-TVMV, a binary vector that includes a cDNA copy of the TVMV genome and regulatory sequences for its plant delivery and expression. Vector components are indicated (bottom); *npt*I, kanamycin resistance gene

A full-length TVMV cDNA clone, under the T7 RNA polymerase promoter (Domier et al. 1989), was used as a control during method validation (Additional file 1: Table S1). The pLX series includes mini T-DNA binary vectors (~ 3.5 kb) that autonomously replicate in *E. coli* and *Agrobacterium*, have plasmid stabilizing features and duplicated left borders to reduce backbone transfer to plants; the pLX vectors have been used in the assembly of plant virus infectious clones for agro-infection (Pasin et al. 2017, 2018; Bao et al. 2020; Klenov and Hudak 2021). A pLX-based vector that includes the cauliflower mosaic virus (CaMV) 35S promoter and nopaline synthase (nos) terminator sequences was linearized by PCR using primers #1F/#1R (Fig. 1a and Additional file 1: Table S2). pXBS7 was used as the template to amplify three cDNA fragments spanning the TVMV genome (Fig. 1a). PCR primers were designed to generate DNA fragments with overlapping termini compatible with GA (Additional file 1: Table S3). cDNA fragments and the linearized vector backbone were subjected to GA using the home-made premix (Table 3). Reactions were transformed into in-house prepared *E. coli* competent cells. Restriction analysis of plasmid DNA purified from selected colonies confirmed the presence of a TVMV-specific fragment in 8 out of the 8 colonies analyzed (Fig. 1b). A plasmid with the correct restriction pattern was designated pLX-TVMV, a 13.6-kb binary vector that includes an intron-free cDNA copy of the full-length TVMV genome. pLX-TVMV includes elements for its stable maintenance in bacterial cells and its *Agrobacterium*-mediated delivery, and regulatory sequences to drive TVMV genome expression in plants (Fig. 1c).

### One-step verification of the infectious clone using high-throughput Illumina sequencing

**Fig. 2:**
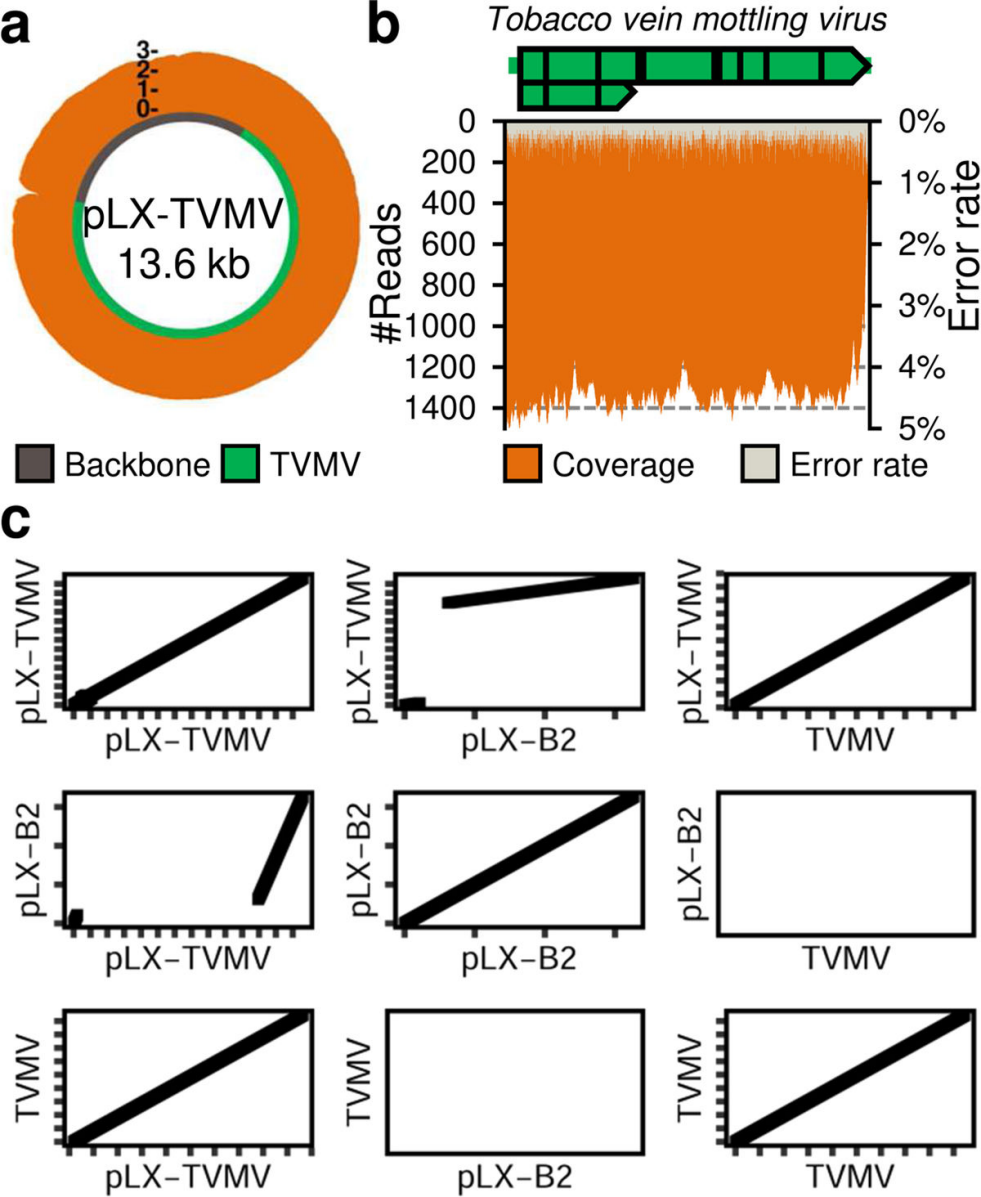
One-step verification of complete *Potyvirus* clone sequence by Illumina sequencing. **a** The sequence of the 13.6-kb pLX-TVMV vector was verified using the Illumina platform and de novo read assembly; the read coverage (log10), vector backbone and TVMV sequences are indicated. **b** The sequencing depth (read number, orange) and error rate (gray) of the TVMV genome assembled are plotted. **c** Dot plots show significant DNA alignments between pLX-TVMV (this study, GenBank: MW027845) and pLX-B2 (GenBank: KY825137) or a TVMV reference genome (GenBank: X04083); axis ticks indicate 1-kb intervals

Once assembled, sequences of infectious clones and DNA constructs in general are commonly verified by the dideoxy chain-termination sequencing method (Sanger). Up to ~ 1-kb sequencing reads are obtained by Sanger; verification of large constructs such as potyvirid cDNA clones thus requires time-consuming primer walking strategies, multiple sequencing reactions, and custom-designed and synthesized oligonucleotides. High-throughput sequencing and read assembly pipelines have been developed to validate synthetic plasmids, including viral clones and vectors (Shapland et al. 2015; Pasin et al. 2018; Saveliev et al. 2018; Gallegos et al. 2020). To overcome the limitations of Sanger sequencing, we used high-throughput sequencing on the Illumina platform for rapid, convenient verification of pLX-TVMV. Sequencing of a paired-end library prepared from ~ 1 μg of purified plasmid recovered the complete sequence of the 13.6-kb pLX-TVMV binary vector with a coverage of > 1000× and an average error rate of 0.16% (Fig. 2a and Additional file 1: Table S4). Given the high sequencing depth and low error rate obtained (Fig. 2b), the pLX-TVMV was de novo assembled using an automated bioinformatic pipeline (see Methods). Sequences of the complete binary vector and its TVMV cDNA clone were deposited to GenBank (accessions MW027845 and MW027846, respectively). An identical sequence was obtained by Illumina sequencing of a second, independent clone of pLX-TVMV (not shown). Sequence alignments were computed between pLX-TVMV and the pLX-B2 vector (GenBank: KY825137) or the TVMV type strain (GenBank: X04083). Dot plots of pLX-TVMV versus pLX-B2 showed a major gap consistent with insertion of the TVMV expression cassette into a vector backbone based on pLX-B2. A diagonal line emerged by plotting the sequences of pLX-TVMV and the TVMV type strain against each other; this continuous match indicated the absence of sequence insertions or deletions in the TVMV cDNA sequence of pLX-TVMV (Fig. 2c). Compared with the accession X04083, the cDNA genomic sequence of pLX-TVMV revealed a 99.8% identity (9454/9475) with 21 point mutations resulting in 10 amino acid changes in the TVMV polyprotein.

### Delivery of the infectious clone assembled to plants by agro-infection

**Fig. 3:**
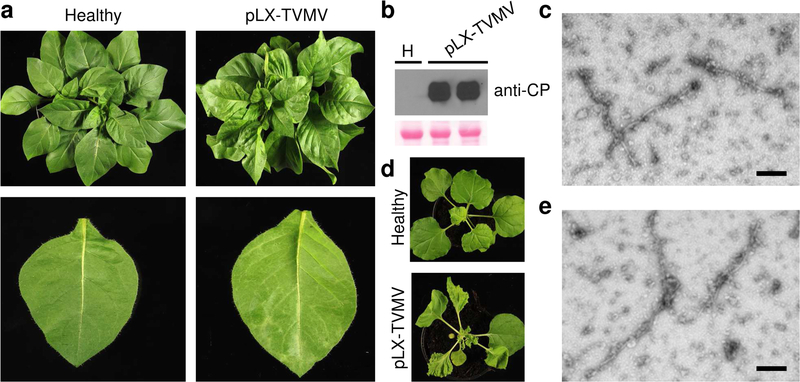
*Agrobacterium*-mediated delivery to plants of an intron-free, pLX-based cDNA clone of tobacco vein mottling virus (TVMV). pLX-TVMV was transformed into *Agrobacterium* and delivered to *Nicotiana* spp. by agro-infection; data were collected after 18 d. **a** Symptoms of the pLX-TVMV-treated *N. clevelandii* plants; untreated plants are shown as a control (Healthy). **b** Viral accumulation was assessed by anti-TVMV coat protein (CP) immunoblotting of upper uninoculated *N. clevelandii* leaves; H, untreated control; Ponceau red-stained blot is shown as a loading control. **c** Transmission electron micrograph of particles observed in infected *N. clevelandii* material; scale bar, 200 nm. **d** Symptoms of the pLX-TVMV-treated *N. benthamiana* plants; healthy, an untreated control. **e** Transmission electron micrograph of particles observed in infected *N. benthamiana* material; scale bar, 200 nm

The host range of TVMV includes *Nicotiana* spp., as well as other species in the family Solanaceae (Sun et al. 1974). The pLX-TVMV binary vector was transformed into *Agrobacterium* cells and delivered to *N. clevelandii* plants to confirm infectivity of the clone assembled. *Agrobacterium* suspensions were infiltrated into plant leaves with a needleless syringe; typical veinbanding symptoms were visible in treated plants at 18 days post-infiltration (Fig. 3a). Total protein extracts from uninoculated leaves were prepared and analyzed by immunoblotting with an antiserum raised against the TVMV coat protein (CP). TVMV CP was detected in samples from the pLX-TVMV-treated plants (Fig. 3b). No symptoms or TVMV CP accumulation were detected in untreated plants (Fig. 3a, b). Leaf deformation was observed in *N. benthamiana* plants agro-inoculated with pLX-TVMV, but not in the control condition (Fig. 3d). Crude extracts from plants agro-inoculated with pLX-TVMV were infectious and were used as the inoculum to launch infection of healthy plants (not shown). Flexuous rods, typical of potyviral particles, were detected in crude extracts of pLX-TVMV-treated plants by electron microscopy (Fig. 3c, e). The collective results (Figs. 1, 2 and 3) confirm the successful assembly of an intron-free, pLX-based infectious clone of an RNA virus and its delivery to plants by agro-infection.

## Discussion

An unprecedented number of plant viruses have been discovered in recent years, and there is increasing demand for methods to study their biology and role in disease development (Massart et al. 2017; Pasin et al. 2019; Villamor et al. 2019; Cieniewicz et al. 2020). We reasoned that home-made reagents would allow convenient and scalable assembly of infectious clones for the biological characterization of viruses, including those discovered by high-throughput sequencing and viral agents of emerging infectious disease. Gibson assembly (Gibson et al. 2009) has been adopted for viral construct assembly and engineering (Siridechadilok et al. 2013; Blawid and Nagata 2015; Pasin et al. 2019). Based on several reports of infectious clone assembly and reverse genetics of viruses (Pasin et al. 2014; Bordat et al. 2015; Suhardiman et al. 2015), we describe the procedures required for home-made preparation of cloning materials for GA. We successfully applied the home-made enzymatic premix and bacterial competent cells in one-step assembly of a T-DNA binary vector that includes the infectious cDNA clone of an RNA virus. Our cloning strategy was streamlined by omission of slow-growing cloning chassis (e.g. yeast) or *ad hoc* engineering steps of viral sequences (e.g. for intron insertion). Cloning used a standard laboratory strain of *E. coli*, and bacterial cells for high-efficiency transformation were prepared in-house without specialized equipment.

High-throughput sequencing is predicted to soon become the routine verification method for synthetic DNA constructs (Shapland et al. 2015; Currin et al. 2019; Gallegos et al. 2020). The full-length sequence of the plasmid assembled was verified using the Illumina platform in a single sequencing reaction that required no custom primers or data analysis pipelines, and avoided time-consuming primer walking strategies. The adoption of Illumina for sequencing of large viral clones as those of potyviruses is facilitated by data processing pipelines that require no or minimal bioinformatics skills (Afgan et al. 2018; Gallegos et al. 2020), as well as by current availability of commercial services with competitive turn-around times and prices (e.g. ~ 35 USD/plasmid at seqWell™, <https://seqwell.com/>, accessed October 20, 2020).

Agro-infection is the most efficient and universal way of delivering plant viruses, and a convenient alternative in terms of cost and scalability to other approaches such as inoculation of DNA plasmids or RNA transcripts. The clone generated was suitable for agro-infection since it was assembled into a plasmid backbone derived from a set of mini T-DNA binary vectors that autonomously and stably replicate in *E. coli* and *Agrobacterium* (Pasin et al. 2017). Finally, the clone infectivity was confirmed based on host symptoms, and immunological detection and visualization of virus particles in agro-inoculated plant samples.

## Conclusions

We described herein convenient home-made cloning materials, Gibson assembly and Illumina verification strategies that were successfully used for one-step assembly and verification of an infectious clone of a large plant virus. We anticipate that the protocols and procedures described herein will support adoption and further development of enhanced methods for characterization of viruses and their role in disease. Our work will streamline the validation of metagenomics and high-throughput sequencing discoveries and guide policy makers in adopting sound strategies to control emerging virus diseases.

## Methods

### Bacterial strains and competent cells

*E. coli* DH10B was used for cloning and plasmid propagation; competent cells were prepared as described (Green and Sambrook 2018). The *Agrobacterium* C58C1–313 strain (Pasin et al. 2017) was used for agro-infection; *Agrobacterium* competent cells were prepared as described (Höfgen and Willmitzer 1988). Unless otherwise indicated, bacteria were grown in lysogeny broth (LB) medium and antibiotics used at final concentrations of 50 mg/L kanamycin and 50 mg/L rifampicin.

### DNA constructs and cloning

pXBS7 and pLX-PPV, a pLX-B2 derivative, have been described (Domier et al. 1989; Pasin et al. 2017) and were used as templates (Additional file 1: Table S1). PCR reactions were performed with Phusion^®^ Hot Start II DNA polymerase (Thermo Fisher Scientific), *Dnp*I treated to remove plasmid templates, and gel purified. The purified DNA fragments were mixed and used in one-step isothermal DNA assembly reactions containing 100 mM Tris-HCl pH 7.5, 10 mM MgCl_2_, 10 mM DTT, 0.2 mM dNTP mix (each), 1 mM NAD^+^, 5% w/v PEG-8000, 0.004 U/μL T5 exonuclease, 0.025 U/μL Phusion^®^ High-Fidelity DNA polymerase, and 4 U/μL *Taq* DNA ligase. Reactions were incubated at 50 °C (1 h) and transformed into chemically-competent *E. coli* DH10B cells; colonies were selected (30 °C, ~ 36 h) onto medium plates supplemented with kanamycin.

### Illumina sequencing

Illumina verification of the complete plasmid sequence was done as described (Pasin et al. 2018). Briefly, plasmid DNA was purified by silica column kits, sheared and used for library preparation. Libraries were sequenced (2 × 150 nt paired-end reads) in an Illumina MiSeq sequencer at the MGH CCIB DNA core facility (U.S.A.; <https://dnacore.mgh.harvard.edu/>, accessed October 20, 2020). Plasmid sequences were determined by de novo read assembly using UltraCycler v1.0 (Brian Seed and Huajun Wang, unpublished); consistency of the assembly was verified by Shovill 1.1.0 using the default options of its Galaxy wrapper (Afgan et al. 2018). Dotplots from DNA sequences were generated using an R package (dotplot <https://github.com/evolvedmicrobe/dotplot>, accessed October 20, 2020).

### Plant materials and virus inoculation

*Nicotiana clevelandii* and *N. benthamiana* plants were grown in a greenhouse (16 h light/8 h dark photoperiod; 19–23 °C temperature regime). pLX-TVMV was transformed into *Agrobacterium* cells by freeze−thawing (Höfgen and Willmitzer 1988); colonies were selected (28 °C, 48–72 h) onto medium plates supplemented with kanamycin and rifampicin. The colonies were used directly, or 20% glycerol bacterial stocks were prepared and placed at − 80 °C for long-term storage. Agro-infection assays were done as described (Pasin et al. 2014). Briefly, fresh *Agrobacterium* colonies or stocks were used to inoculate 1 mL LB supplemented with kanamycin and rifampicin, and incubated for 24–48 h at 28 °C, 250 rpm; a 100 μL aliquot was then inoculated to 5 mL LB supplemented with kanamycin and rifampicin, and incubated for 12–18 h at 28 °C, 250 rpm. Cultures were centrifuged, bacteria harvested, washed and incubated for 3 h in 10 mM 2-(*N*-morpholino) ethane sulfonic acid, pH 5.5, 10 mM MgCl_2_ and 150 μM acetosyringone at room temperature in the dark. The OD_600_ of the suspensions was adjusted to 0.5, and syringe-infiltrated into two young leaves of 3-week-old plants.

### Protein and virion detection

Total protein extracts from plant samples were prepared and resolved by SDS-PAGE, as described (Pasin et al. 2020). Immuno-detection was conducted using a rabbit anti-TVMV coat protein serum (Domier et al. 1989) as the primary antibody, and a peroxidase goat anti-rabbit IgG (Jackson ImmunoResearch Laboratories) as the conjugate. Virion micrographs were obtained by immunosorbent electron microscopy as described (Valli et al. 2014). Briefly, plant extracts were prepared in 5 mM Tris-HCl (pH 7.5), 150 mM NaCl, 2.5 mM DTT, and incubated with collodion-coated carbon-stabilized copper grids precoated with the anti-TVMV CP serum. Grids were negative-stained with 2% uranyl acetate and observed in a transmission electron microscope (JEM 1011, Jeol); images were taken with the ES1000W Erlangshen CCD camera (Gatan). ImageJ (Schneider et al. 2012) was used for image processing.

## Supplementary Material

1677486_SuppFiles

## Figures and Tables

**Table 1:** Reagents for preparation of the home-made Gibson assembly (GA) enzymatic premix

Reagent	Provider	Identifier
Tris(hydroxymethyl)aminomethane (Tris base)	Sigma-Aldrich	T1503
Poly(ethylene glycol) MW ~ 8000 (PEG-8000)	MP Biomedicals	195445
MgCl_2_	Sigma-Aldrich	442611
Dithiothreitol (DTT)	Promega	V3151
dNTP mix	Thermo Fisher Scientific	R1121
β-Nicotinamide adenine dinucleotide (NAD^+^)	New England Biolabs	B9007S
T5 exonuclease	New England Biolabs	M0663S
Phusion^®^ High-Fidelity DNA polymerase	New England Biolabs	M0530S
*Taq* DNA ligase	New England Biolabs	M0208S
Nuclease-free distilled H_2_O (n.f. dH_2_O)^a^	–	–

a Autoclaved Milli-Q^®^ ultrapure water

**Table 2:** GA reaction buffer (3.33×, 350 μL)

Reagent	Stock concentration	Volume	Final concentration
Tris-HCl, pH 7.5^a^	1000 mM	115.5 μL	330 mM
MgCl_2_^a^	2000 mM	5.8 μL	33 mM
DTT	1000 mM	11.6 μL	33 mM
dNTP mix	25 mM (each)	9.2 μL	0.66 mM
NAD^+^	50 mM	23.1 μL	3.30 mM
PEG-8000^b^	35%	165.0 μL	16.50%
n.f. dH_2_O	–	19.8 μL	–
Total volume	–	350.0 μL	–

a Autoclaved solution

b 0.22-μm filtered solution

**Table 3:** GA enzymatic premix (2×, 125 μL)

Reagent	Stock concentration	Volume	Final concentration
GA reaction buffer	3.33×	75.8 μL	2×
T5 exonuclease^a^	0.1 U/μL	10.0 μL	0.008 U/μL
Phusion^®^ High-Fidelity DNA polymerase	2 U/μL	3.1 μL	0.05 U/μL
*Taq* DNA ligase	40 U/μL	25.0 μL	8 U/μL
n.f. dH_2_O	–	11.1 μL	–
Total volume	–	125.0 μL	–

a The 10 U/μL provider enzyme is diluted in n.f. dH_2_O to 0.1 U/μL before use

## Ready to submit your research? Choose BMC and benefit from:

- fast, convenient online submission
- thorough peer review by experienced researchers in your field
- repid publication on acceptance
- support for research data, including large and complex data types
- gold Open Access which fosters wider collaboration and increased citations
- maximum visibility for your research: over 100M website views per year

## Abbreviations

CaMV: Cauliflower mosaic virus
CP: Coat protein
*E. coli*: *Escherichia coli*
GA: Gibson assembly
LB: Lysogeny broth
LMV: Lettuce mosaic virus
*N. benthamiana*: *Nicotiana benthamiana*
*N. clevelandii*: *Nicotiana clevelandii*
nos: Nopaline synthase
PPV: Plum pox virus
TVMV: Tobacco vein mottling virus
